# 1-(2-Fluoro­phen­yl)-3-(2,4,6-trimeth­oxy­phen­yl)prop-2-en-1-one

**DOI:** 10.1107/S1600536812043139

**Published:** 2012-10-20

**Authors:** M. Prabuswamy, S. Madan Kumar, D. Bhuvaneshwar, Ch. S. S. S. Murthy, N. K. Lokanath

**Affiliations:** a Department of Studies in Physics, Manasagangotri, University of Mysore, Mysore 570 006, India; b Department of Nanotechnology, School of Interdisciplinary Courses, Noorul Islam Centre for Higher Education, Kumarcoil, Kanyakumari 629 180, India

## Abstract

In the title compound, C_18_H_17_FO_4_, the dihedral angle between the aromatic rings is 32.29 (8)°. The C atoms of the meth­oxy groups deviate from their attached ring plane by 0.018 (2), −0.006 (2) and −0.094 (2) Å. In the crystal, C—H⋯O hydrogen bonds link the mol­ecules into *C*(6) [001] chains.

## Related literature

For the synthesis and properties of the title compound, see: Rimal *et al.* (2012 ▶). For a related structure, see: Jasinski *et al.* (2009 ▶).
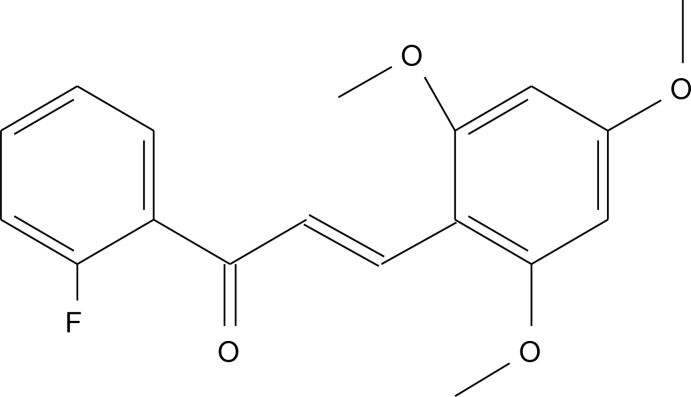


## Experimental

### 

#### Crystal data

C_18_H_17_FO_4_
*M* *_r_* = 316.32
Monoclinic, 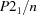

*a* = 7.0927 (3) Å
*b* = 25.9711 (11) Å
*c* = 8.7487 (4) Å
β = 91.584 (4)°
*V* = 1610.94 (12) Å^3^
*Z* = 4
Mo *K*α radiation
μ = 0.10 mm^−1^
*T* = 296 K
0.22 × 0.22 × 0.20 mm

#### Data collection

Oxford Diffraction Xcalibur Eos CCD diffractometer
16217 measured reflections
3057 independent reflections
1946 reflections with *I* > 2σ(*I*)
*R*_int_ = 0.037

#### Refinement

*R*[*F* ^2^ > 2σ(*F* ^2^)] = 0.039
*wR*(*F* ^2^) = 0.100
*S* = 1.00
3057 reflections
212 parameters
H-atom parameters constrained
Δρ_max_ = 0.17 e Å^−3^
Δρ_min_ = −0.14 e Å^−3^

### 

Data collection: *CrysAlis PRO* (Oxford Diffraction, 2009 ▶); cell refinement: *CrysAlis PRO*; data reduction: *CrysAlis PRO*; program(s) used to solve structure: *SHELXS97* (Sheldrick, 2008 ▶); program(s) used to refine structure: *SHELXL97* (Sheldrick, 2008 ▶); molecular graphics: *Mercury* (Macrae *et al.*, 2006 ▶); software used to prepare material for publication: *SHELXL97* and *Mercury*.

## Supplementary Material

Crystal structure: contains datablock(s) global, I. DOI: 10.1107/S1600536812043139/hb6965sup1.cif

Structure factors: contains datablock(s) I. DOI: 10.1107/S1600536812043139/hb6965Isup2.hkl

Supplementary material file. DOI: 10.1107/S1600536812043139/hb6965Isup3.cml

Additional supplementary materials:  crystallographic information; 3D view; checkCIF report

## Figures and Tables

**Table 1:** Hydrogen-bond geometry (Å, °)

*D*—H⋯*A*	*D*—H	H⋯*A*	*D*⋯*A*	*D*—H⋯*A*
C2—H2⋯O1^i^	0.93	2.44	3.338 (2)	162

Symmetry code: (i) 
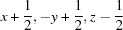
.

**Fig. 1.**
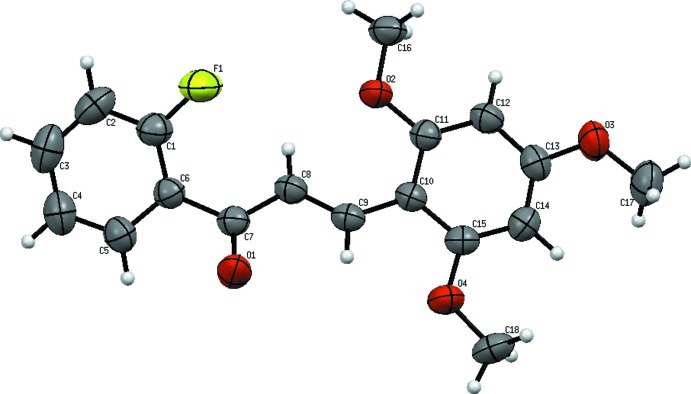
ORTEP diagram of the title compound with 50% probability ellipsoids.

**Fig. 2.**
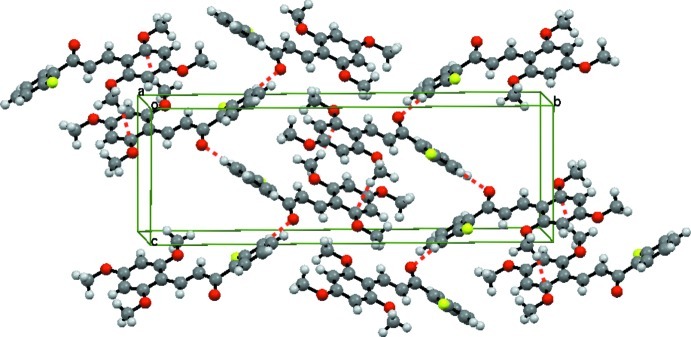
Packing diagram of the title compound, viewed along the crystallographic a axis. C—H···O hydrogen bonds are indicated by dashed lines.

## supplementary crystallographic information

### Comment

As part of our ongoing studies of chalcones with possible biological activity (Rimal *et al.*, 2012), we now describe the crystal structure of 1-(2-fluorophenyl)-3-(2,4,6-trimethoxyphenyl)prop-2-en-1-one, (I).

The *ORTEP* drawing of the title molecule is as shown in Fig. 1. The dihedral angle between the aromatic rings of 2-flurophenyl and trimethoxy phenyl group is 32.29 (8)°. Mean plane of prop-2-ene-1 one moiety makes an angle of 37.65 (9)° with 2-flurophenyl and 11.64 (9)° with trimethoxyphenyl moiety. The overall geometry of the title compound is similar to that of (2E)-1-(4-fluorophenyl)-3-(3,4,5-trimethoxyphenyl)prop-2-en-1-one (Jasinski *et al.*, 2009).

The molecules are connected by C—H···O interactions (Fig. 2).

### Experimental

The title compound was synthesized as per the procedure reported in the literature (Rimal *et al.*, 2012). The final product was obtained by recrystallization using aqueous ethyl acetate: methanol as a solvent. Slow evaporation method yielded brown blocks.

### Refinement

All the hydrogen atoms of the compound are fixed geometrically (C—H= 0.93–0.97 Å) and allowed to ride on their parent atoms.

### Crystal data

**Table:**

C_18_H_17_FO_4_	*F*(000) = 664
*M**_r_* = 316.32	*D*_x_ = 1.304 Mg m^−^^3^
Monoclinic, *P*2_1_/*n*	Mo *K*α radiation, λ = 0.71073 Å
Hall symbol: -P 2yn	Cell parameters from 3057 reflections
*a* = 7.0927 (3) Å	θ = 2.5–25.7°
*b* = 25.9711 (11) Å	µ = 0.10 mm^−^^1^
*c* = 8.7487 (4) Å	*T* = 296 K
β = 91.584 (4)°	Block, brown
*V* = 1610.94 (12) Å^3^	0.22 × 0.22 × 0.20 mm
*Z* = 4	

### Data collection

**Table:**

Oxford Diffraction Xcalibur Eos CCD diffractometer	1946 reflections with *I* > 2σ(*I*)
Radiation source: fine-focus sealed tube	*R*_int_ = 0.037
Graphite monochromator	θ_max_ = 25.7°, θ_min_ = 2.5°
Detector resolution: 16.0839 pixels mm^-1^	*h* = −8→8
ω scans	*k* = −31→30
16217 measured reflections	*l* = −10→10
3057 independent reflections	

### Refinement

**Table:**

Refinement on *F*^2^	Secondary atom site location: difference Fourier map
Least-squares matrix: full	Hydrogen site location: inferred from neighbouring sites
*R*[*F*^2^ > 2σ(*F*^2^)] = 0.039	H-atom parameters constrained
*wR*(*F*^2^) = 0.100	*w* = 1/[σ^2^(*F*_o_^2^) + (0.0497*P*)^2^] where *P* = (*F*_o_^2^ + 2*F*_c_^2^)/3
*S* = 1.00	(Δ/σ)_max_ < 0.001
3057 reflections	Δρ_max_ = 0.17 e Å^−^^3^
212 parameters	Δρ_min_ = −0.14 e Å^−^^3^
0 restraints	Extinction correction: *SHELXL97* (Sheldrick, 2008), FC^*^=KFC[1+0.001XFC^2^Λ^3^/SIN(2Θ)]^-1/4^
Primary atom site location: structure-invariant direct methods	Extinction coefficient: 0.023 (2)

### Special details

**Table:**

Geometry. Bond distances, angles *etc*. have been calculated using the rounded fractional coordinates. All su's are estimated from the variances of the (full) variance-covariance matrix. The cell e.s.d.'s are taken into account in the estimation of distances, angles and torsion angles
Refinement. Refinement on *F*^2^ for ALL reflections except those flagged by the user for potential systematic errors. Weighted *R*-factors *wR* and all goodnesses of fit *S* are based on *F*^2^, conventional *R*-factors *R* are based on *F*, with *F* set to zero for negative *F*^2^. The observed criterion of *F*^2^ > σ(*F*^2^) is used only for calculating -*R*-factor-obs *etc*. and is not relevant to the choice of reflections for refinement. *R*-factors based on *F*^2^ are statistically about twice as large as those based on *F*, and *R*-factors based on ALL data will be even larger.

### Fractional atomic coordinates and isotropic or equivalent isotropic displacement parameters (Å^2^)

**Table:**

	*x*	*y*	*z*	*U*_iso_*/*U*_eq_	
F1	0.47580 (14)	0.21236 (4)	0.09672 (13)	0.0862 (5)	
O1	0.05893 (17)	0.14446 (4)	0.32501 (13)	0.0687 (4)	
O2	0.65872 (14)	0.07225 (4)	0.09354 (13)	0.0565 (4)	
O3	0.81263 (17)	−0.10017 (4)	0.20765 (13)	0.0681 (5)	
O4	0.24772 (16)	−0.02139 (4)	0.39762 (13)	0.0641 (4)	
C1	0.2939 (2)	0.22710 (6)	0.06805 (19)	0.0569 (7)	
C2	0.2679 (3)	0.27123 (7)	−0.0164 (2)	0.0709 (8)	
C3	0.0855 (4)	0.28686 (7)	−0.0490 (2)	0.0775 (9)	
C4	−0.0645 (3)	0.25900 (7)	0.0031 (2)	0.0724 (8)	
C5	−0.0318 (2)	0.21601 (6)	0.09185 (19)	0.0583 (6)	
C6	0.1499 (2)	0.19858 (6)	0.12600 (17)	0.0463 (5)	
C7	0.1721 (2)	0.15104 (6)	0.22354 (17)	0.0464 (5)	
C8	0.3191 (2)	0.11421 (6)	0.19021 (17)	0.0471 (5)	
C9	0.3266 (2)	0.06858 (5)	0.26294 (17)	0.0444 (5)	
C10	0.4532 (2)	0.02558 (5)	0.24723 (16)	0.0422 (5)	
C11	0.6197 (2)	0.02680 (6)	0.16292 (16)	0.0448 (5)	
C12	0.7362 (2)	−0.01548 (6)	0.15323 (17)	0.0516 (6)	
C13	0.6887 (2)	−0.06078 (6)	0.22712 (18)	0.0510 (6)	
C14	0.5274 (2)	−0.06425 (6)	0.31166 (18)	0.0519 (6)	
C15	0.4117 (2)	−0.02141 (6)	0.32072 (16)	0.0463 (6)	
C16	0.8256 (2)	0.07605 (6)	0.00797 (19)	0.0596 (7)	
C17	0.7766 (3)	−0.14799 (6)	0.2812 (2)	0.0782 (8)	
C18	0.1869 (3)	−0.06735 (6)	0.4707 (2)	0.0699 (7)	
H2	0.37020	0.29000	−0.05050	0.0850*	
H3	0.06380	0.31650	−0.10660	0.0930*	
H4	−0.18730	0.26920	−0.02150	0.0870*	
H5	−0.13400	0.19810	0.13000	0.0700*	
H8	0.40860	0.12220	0.11820	0.0570*	
H9	0.23470	0.06400	0.33550	0.0530*	
H12	0.84580	−0.01370	0.09760	0.0620*	
H14	0.49710	−0.09470	0.36140	0.0620*	
H16A	0.93360	0.07030	0.07430	0.0890*	
H16B	0.83310	0.10980	−0.03650	0.0890*	
H16C	0.82270	0.05060	−0.07170	0.0890*	
H17A	0.77770	−0.14300	0.39000	0.1170*	
H17B	0.87250	−0.17240	0.25570	0.1170*	
H17C	0.65550	−0.16080	0.24760	0.1170*	
H18A	0.16980	−0.09410	0.39580	0.1050*	
H18B	0.06970	−0.06100	0.51960	0.1050*	
H18C	0.28040	−0.07790	0.54580	0.1050*	

### Atomic displacement parameters (Å^2^)

**Table:**

	*U*^11^	*U*^22^	*U*^33^	*U*^12^	*U*^13^	*U*^23^
F1	0.0529 (7)	0.1002 (8)	0.1054 (9)	−0.0147 (6)	0.0030 (6)	0.0333 (7)
O1	0.0699 (8)	0.0614 (7)	0.0766 (8)	0.0155 (6)	0.0370 (7)	0.0142 (6)
O2	0.0464 (7)	0.0508 (7)	0.0734 (8)	0.0010 (5)	0.0218 (6)	0.0077 (6)
O3	0.0714 (9)	0.0548 (7)	0.0788 (8)	0.0200 (6)	0.0136 (7)	0.0074 (6)
O4	0.0553 (8)	0.0594 (7)	0.0789 (8)	−0.0048 (6)	0.0233 (6)	0.0108 (6)
C1	0.0501 (12)	0.0633 (11)	0.0573 (11)	−0.0054 (9)	0.0000 (9)	0.0046 (9)
C2	0.0886 (16)	0.0605 (12)	0.0639 (12)	−0.0176 (11)	0.0057 (11)	0.0126 (10)
C3	0.1100 (19)	0.0544 (12)	0.0676 (13)	0.0113 (13)	−0.0069 (13)	0.0107 (10)
C4	0.0753 (15)	0.0611 (12)	0.0803 (14)	0.0190 (11)	−0.0039 (11)	0.0038 (10)
C5	0.0552 (12)	0.0542 (10)	0.0659 (11)	0.0074 (9)	0.0078 (9)	−0.0036 (9)
C6	0.0465 (10)	0.0444 (9)	0.0482 (9)	−0.0005 (8)	0.0055 (8)	−0.0024 (7)
C7	0.0435 (9)	0.0471 (9)	0.0490 (9)	−0.0032 (8)	0.0067 (8)	−0.0024 (7)
C8	0.0405 (9)	0.0525 (10)	0.0488 (9)	0.0013 (8)	0.0083 (7)	0.0004 (8)
C9	0.0383 (9)	0.0505 (9)	0.0447 (9)	−0.0028 (7)	0.0059 (7)	−0.0032 (7)
C10	0.0369 (9)	0.0464 (9)	0.0435 (8)	−0.0006 (7)	0.0024 (7)	−0.0016 (7)
C11	0.0427 (9)	0.0456 (9)	0.0462 (9)	−0.0009 (8)	0.0021 (7)	−0.0007 (7)
C12	0.0444 (10)	0.0573 (10)	0.0536 (10)	0.0035 (8)	0.0095 (8)	−0.0001 (8)
C13	0.0515 (11)	0.0504 (10)	0.0510 (10)	0.0083 (8)	−0.0013 (8)	−0.0044 (8)
C14	0.0576 (11)	0.0445 (9)	0.0535 (10)	−0.0025 (8)	0.0013 (9)	0.0026 (8)
C15	0.0419 (10)	0.0514 (10)	0.0457 (9)	−0.0057 (8)	0.0045 (8)	−0.0005 (7)
C16	0.0488 (11)	0.0609 (11)	0.0700 (12)	−0.0068 (8)	0.0182 (9)	−0.0010 (9)
C17	0.0957 (16)	0.0518 (11)	0.0871 (14)	0.0165 (11)	0.0049 (12)	0.0052 (10)
C18	0.0680 (13)	0.0693 (12)	0.0732 (13)	−0.0206 (10)	0.0182 (10)	0.0127 (10)

### Geometric parameters (Å, º)

**Table:**

F1—C1	1.3624 (18)	C12—C13	1.388 (2)
O1—C7	1.2251 (19)	C13—C14	1.382 (2)
O2—C11	1.3593 (19)	C14—C15	1.386 (2)
O2—C16	1.4213 (18)	C2—H2	0.9300
O3—C13	1.3625 (19)	C3—H3	0.9300
O3—C17	1.4251 (19)	C4—H4	0.9300
O4—C15	1.3596 (18)	C5—H5	0.9300
O4—C18	1.427 (2)	C8—H8	0.9300
C1—C2	1.373 (2)	C9—H9	0.9300
C1—C6	1.370 (2)	C12—H12	0.9300
C2—C3	1.378 (3)	C14—H14	0.9300
C3—C4	1.375 (3)	C16—H16A	0.9600
C4—C5	1.376 (2)	C16—H16B	0.9600
C5—C6	1.391 (2)	C16—H16C	0.9600
C6—C7	1.507 (2)	C17—H17A	0.9600
C7—C8	1.451 (2)	C17—H17B	0.9600
C8—C9	1.345 (2)	C17—H17C	0.9600
C9—C10	1.4419 (19)	C18—H18A	0.9600
C10—C11	1.410 (2)	C18—H18B	0.9600
C10—C15	1.414 (2)	C18—H18C	0.9600
C11—C12	1.378 (2)		
			
C11—O2—C16	118.58 (11)	C3—C2—H2	121.00
C13—O3—C17	118.26 (13)	C2—C3—H3	120.00
C15—O4—C18	119.66 (13)	C4—C3—H3	120.00
F1—C1—C2	116.49 (15)	C3—C4—H4	120.00
F1—C1—C6	119.46 (14)	C5—C4—H4	120.00
C2—C1—C6	124.04 (15)	C4—C5—H5	119.00
C1—C2—C3	117.94 (18)	C6—C5—H5	119.00
C2—C3—C4	120.46 (17)	C7—C8—H8	120.00
C3—C4—C5	119.62 (19)	C9—C8—H8	120.00
C4—C5—C6	121.75 (15)	C8—C9—H9	115.00
C1—C6—C5	116.13 (14)	C10—C9—H9	115.00
C1—C6—C7	125.82 (13)	C11—C12—H12	120.00
C5—C6—C7	118.05 (13)	C13—C12—H12	120.00
O1—C7—C6	117.67 (13)	C13—C14—H14	121.00
O1—C7—C8	122.87 (14)	C15—C14—H14	121.00
C6—C7—C8	119.41 (13)	O2—C16—H16A	109.00
C7—C8—C9	120.33 (13)	O2—C16—H16B	109.00
C8—C9—C10	130.82 (14)	O2—C16—H16C	109.00
C9—C10—C11	124.42 (13)	H16A—C16—H16B	110.00
C9—C10—C15	119.15 (13)	H16A—C16—H16C	109.00
C11—C10—C15	116.43 (13)	H16B—C16—H16C	110.00
O2—C11—C10	115.91 (13)	O3—C17—H17A	109.00
O2—C11—C12	122.32 (13)	O3—C17—H17B	109.00
C10—C11—C12	121.77 (14)	O3—C17—H17C	110.00
C11—C12—C13	119.53 (13)	H17A—C17—H17B	109.00
O3—C13—C12	114.35 (13)	H17A—C17—H17C	109.00
O3—C13—C14	124.35 (14)	H17B—C17—H17C	109.00
C12—C13—C14	121.30 (14)	O4—C18—H18A	109.00
C13—C14—C15	118.58 (14)	O4—C18—H18B	109.00
O4—C15—C10	114.60 (13)	O4—C18—H18C	109.00
O4—C15—C14	122.99 (13)	H18A—C18—H18B	110.00
C10—C15—C14	122.39 (13)	H18A—C18—H18C	109.00
C1—C2—H2	121.00	H18B—C18—H18C	110.00
			
C16—O2—C11—C10	179.43 (13)	O1—C7—C8—C9	5.6 (2)
C16—O2—C11—C12	0.1 (2)	C6—C7—C8—C9	−171.60 (14)
C17—O3—C13—C12	179.14 (14)	C7—C8—C9—C10	178.00 (14)
C17—O3—C13—C14	−1.2 (2)	C8—C9—C10—C11	10.4 (3)
C18—O4—C15—C10	177.28 (13)	C8—C9—C10—C15	−169.93 (15)
C18—O4—C15—C14	−1.3 (2)	C9—C10—C11—O2	0.1 (2)
F1—C1—C2—C3	179.23 (15)	C9—C10—C11—C12	179.39 (14)
C6—C1—C2—C3	−2.2 (3)	C15—C10—C11—O2	−179.62 (12)
F1—C1—C6—C5	−179.97 (13)	C15—C10—C11—C12	−0.3 (2)
F1—C1—C6—C7	0.8 (2)	C9—C10—C15—O4	1.86 (19)
C2—C1—C6—C5	1.5 (2)	C9—C10—C15—C14	−179.54 (14)
C2—C1—C6—C7	−177.78 (16)	C11—C10—C15—O4	−178.45 (12)
C1—C2—C3—C4	0.5 (3)	C11—C10—C15—C14	0.2 (2)
C2—C3—C4—C5	1.8 (3)	O2—C11—C12—C13	179.81 (13)
C3—C4—C5—C6	−2.5 (3)	C10—C11—C12—C13	0.5 (2)
C4—C5—C6—C1	0.9 (2)	C11—C12—C13—O3	179.05 (13)
C4—C5—C6—C7	−179.77 (15)	C11—C12—C13—C14	−0.6 (2)
C1—C6—C7—O1	144.10 (16)	O3—C13—C14—C15	−179.15 (14)
C1—C6—C7—C8	−38.5 (2)	C12—C13—C14—C15	0.5 (2)
C5—C6—C7—O1	−35.2 (2)	C13—C14—C15—O4	178.24 (14)
C5—C6—C7—C8	142.22 (15)	C13—C14—C15—C10	−0.2 (2)

### Hydrogen-bond geometry (Å, º)

**Table:**

*D*—H···*A*	*D*—H	H···*A*	*D*···*A*	*D*—H···*A*
C2—H2···O1^i^	0.93	2.44	3.338 (2)	162

Symmetry code: (i) *x*+1/2, −*y*+1/2, *z*−1/2.
